# Sex determination and sex chromosome evolution in land plants

**DOI:** 10.1098/rstb.2021.0210

**Published:** 2022-05-09

**Authors:** Susanne S. Renner, Niels A. Müller

**Affiliations:** ^1^ Department of Biology, Washington University, Saint Louis, MO 63130, USA; ^2^ Thünen-Institute of Forest Genetics, Sieker Landstrasse 2, 22927 Grosshansdorf, Germany

**Keywords:** Animal sex determination systems, plant sex determination systems, dioecy pathways, dosage compensation, key open questions

## Abstract

Linnaeus's very first opus, written when he was 22 years old, dealt with the analogy that exists between plants and animals in how they ‘propagate their species’, and a revised version with a plate depicting the union of male and female *Mercurialis annua* plants became a foundational text on the sexuality of plants. The question how systems with separate males and females have evolved in sedentary organisms that appear ancestrally bisexual has fascinated biologists ever since. The phenomenon, termed dioecy, has important consequences for plant reproductive success and is of commercial interest since it affects seed quality and fruit production. This theme issue presents a series of articles that synthesize and challenge the current understanding of how plants achieve dioecy. The articles deal with a broad set of taxa, including *Coccinia*, *Ginkgo*, *Mercurialis*, *Populus*, *Rumex* and *Silene*, as well as overarching topics, such as the field's terminology, analogies with animal sex determination systems, evolutionary pathways to dioecy, dosage compensation, and the longevity of the two sexes. In this introduction, we focus on four topics, each addressed by several articles from different angles and with different conclusions. Our highlighting of unclear or controversial issues may help future studies to build on the current understanding and to ask new questions that will expand our knowledge of plant sexual systems.

This article is part of the theme issue ‘Sex determination and sex chromosome evolution in land plants’.

## 1. Introduction

Why and how did sedentary organisms that ancestrally appear capable of producing both male and female gametes evolve genetically determined systems that suppress the production of the gametes of one sex, while permitting that of the opposite sex? This 300-year-old question has been surprisingly difficult to answer. Considerable progress has been made over the past few years, however, due to genomic work, including chromosome-level genome assemblies of non-model species—which most dioecious plants are—and the possibility of resolving maternal and paternal haplotypes in diploid organisms. Together, these methods have provided insights into the precise chromosomal location of genes involved in the suppression or promotion of male or female gamete-producing structures. This special issue presents a series of articles that advance the current understanding of how plants achieve dioecy. To provide a unifying framework, we focus on four themes that each is addressed by several articles from different angles and with different conclusions (table 1).

**Table 1.** Key questions about the evolution and mechanistic function of sex determination and sex chromosomes in land plants, with relevant contributions in this set of articles.

	level	question	references
a	ultimate	Do sex determination systems share important properties between embryophytes and animals (Holozoa)? Conversely, what are key differences in how animals and embryophytes regulate gonochory and dioecy? Dioecy is derived in embryophyte gametophytes and perhaps in sporophytes, but does this result in generally younger sex chromosomes in plants? Does a sex difference in longevity, related to heteromorphic sex chromosomes, exist in both animals and plants? Is there a difference in the evolution of dosage compensation between animals and plants?	Mank [1] Charlesworth [2] Cronk [3,4] Marais & Lemaitre [5] Muyle *et al*. [6]
b	ultimate	Which pathways to dioecy are expected to result in single-gene and which in two-gene systems? Are there plant systems with single-gene control of separate-sexedness? In flowering plants, when is a pathway to dioecy from gynodioecy, the coexistence of perfect-flowered and pistillate individuals [7], more important, and when a pathway from monoecy, the coexistence of individuals that all bear a mix of male and female flowers?	Charlesworth [2] Cronk [3,4] Gong & Filatov [8] Mank [1] Zluvova *et al*. [9]
c	proximate	What explains the size of the non-recombining region in embryophytes? Do non-recombining regions expand over evolutionary time?	Janousek *et al*. [10] Charlesworth [2] Gong & Filatov [8]
d	proximate	How can we efficiently identify and characterize sex-determining regions? What kind of genes function as sex determinants in dioecious plants? Which molecular pathways underlie the signalling from sex-determining genes to gamete-producing structures?	Leite Montalvão *et al*. [11] Gong & Filatov [8] Janousek *et al.* [10] Rifkin *et al*. [12]

From its very beginning, research into plant sexual systems was inspired and influenced by research on animal sexual systems. Thus, Linnaeus's first-ever paper, a handwritten pamphlet from 1729, which he later revised as a doctoral thesis, deals with the great analogy that exists between animals and plants ‘smitten by love’ [13]. The accompanying plate, chosen for the cover of this theme issue, shows a male and a female individual of *Mercurialis annua* ‘united by love’ and with the wind carrying the male's pollen to the female. Linnaeus argued that just as no chicken hatches unless a rooster has been with the hen, also in plants no seed will arise unless pollen has arrived on a stigma. This was written roughly 100 years before the discovery of the alternation of generations in all embryophytes, a phenomenon that makes comparisons of sexual systems between higher plants and animals if not more problematic at least more complex [3].

Discoveries in animals also led to the discovery of plant sex chromosomes. Microscopically dimorphic sex-specific chromosomes were discovered in insects, with Nettie Stevens's 1905 work especially important because she was the first to concretely show that the Y chromosome was involved in sex determination and that sex itself was a Mendelian trait [14]. It can be argued, however, that Correns's [15] demonstration of sex determination being a Mendelian trait, for which he used experimental crossings of two species of *Bryonia* (Cucurbitaceae), preceded the insect work by 2 years, a fact that did not always sit well with zoologists [16]. There were no sex differences in the *Bryonia* chromosomes though. The first plant sex chromosomes seen through a microscope instead were those of a liverwort [17], which Charles Allen looked for because of the distinct X/Y chromosomes found in insects.

Allen [16, p. 101] was prescient about another similarity between plant and animal sexual systems: ‘Since zoological writers have shown neither undue modesty nor excessive caution in treating of botanical phenomena, one suggestion will be ventured. […] Wherever hermaphroditism, intersexuality or sex-reversal occur—and these are now recognized as widespread phenomena in several metazoan phyla—the potentialities for the production of the characters of both sexes must reside in each individual. The more or less sharp differentiation of male and female individuals, the genetics of sex, and the occurrence of the X/Y chromosome mechanism, [are] all parallel to conditions noted in angiosperms.’ The topic of sexual lability in dioecious species is taken up in this special issue by Käfer *et al.* [18], who find that the occurrence of lability (i.e. production of male and female gametes in single individuals) is not generally related to the number of sex-linked genes or the age of the non-recombining region.

That plant and animal systems of sex determination may have more in common than even Allen [16] suspected is apparent from a recent theme issue of the *Philosophical Transactions B* devoted to vertebrate sexual systems, whose editors conclude that, ‘This group encompasses lineages without sex chromosomes, such as hermaphrodites and species with environmental sex determination, as well as those with sex chromosomes at different stages of differentiation, which makes vertebrates an ideal model system for comparative studies’ [19]. This statement is just as applicable to plants.

## 2. Key topics addressed in this special issue

### (a) The evolutionary differences between animal and plant separate-sexed systems

Given this background, a main area of conceptual discussion is whether the way higher plants and animals regulate their sexual specialization is ‘all that different’ [1]. Highly divergent sex chromosomes appear to be the exception in both animals (the clade Holozoa) and land plants (the clade Embryophyta), while the majority of separate-sexed animals and plants has visually non-dimorphic chromosomes [1,20]. What generalizable differences between animal and plant sex chromosomes or sex-determining systems remain? Is it that sex chromosomes of plants are generally younger than those of animals because dioecy is often a derived trait in plants, while gonochorism (separate sexes) may be ancestral in animals [1,5,6,12]? This argument reflects pre-phylogenetic thinking. It is now clear that there have been numerous transitions in Holozoa (using either Ctenophora or Porifera as outgroups) from separate sexes to hermaphroditism, sometimes followed by the re-evolution of gonochory, although the ancestral condition, a billion years ago, may indeed have been gonochory [21]. In most animals, their motility enables an active mating behaviour and efficient mate-searching system, and they can therefore afford to have separate sexes, while the sessile lifestyle of embryophytes may favour species in which all individuals can produce offspring, which is achieved by hermaphroditism ([22] for a review of the arguments). The ancestral condition of the land plant gametophyte (i.e. the multicellular haploid stage in the life cycle) is difficult to infer because in green algae, the paraphyletic group from which land plants evolved, the gametophyte in some species is unisexual, producing either large (female) or small (male) gametes, but in other species, it instead produces gametes that are not distinguishable as male or female. The best-studied fossil gametophytes of 400 million-year-old land plants are unisexual [23]. Given the sparse empirical data and the time spans in question, broad statements about the ancestral sexual system of ‘animals’ or ‘plants’ are therefore currently hard to justify. Also, as Rifkin *et al.* [12] point out, rates of recombination can vary between species, between and within chromosomes and between male and female meiosis in both dioecious/gonochoric and hermaphroditic species, making generalization even more difficult.

One difference between animals and plants is the greater potential for haploid selection in plants compared to animals [1,24]. Cronk [4,20] pinpoints this to primary selection (the first filtering of the products of meiosis) being via gametes in diplontic animals, but via gametophyte organisms in embryophytes (because of their generation cycling). Another difference is that the developing male germ cells in the testes of vertebrates are linked (forming a syncytum) throughout differentiation until the moment when an individual sperm is released. This sets up the condition for strong haploid selection among male gametes in animals, which has no equivalent in plants. Male animals have therefore evolved to mask the alleles in their gametes to a large extent by sharing mRNA and protein products among developing sperm within a large syncytium with a common cytoplasm.

Another difference between animals and plants might be the so-called sex gap in longevity (more precisely, a difference in the median age distributions of males and females), referring to the observation that male and female animals can display markedly different longevity, which apparently relates to sex chromosomes, with X-hemizygosity and toxicity of the Y chromosomes being the proposed mechanisms. The novel question whether this ‘gap’ also occurs in dioecious plants with sex chromosomes is addressed by Marais and Lemaitre [5], whose results tend to support the correlation also in plants, although empirical cases are few and statistical power is therefore limited. The small number of ZW systems in plants and the absence of any microscopically heteromorphic sex chromosomes in such systems so far preclude the detection of possible toxicity of W chromosomes.

Lastly, Muyle *et al.* [6] review the multiple evolutionary theories that have been proposed to explain dosage compensation patterns in eukaryotes with XY or ZW sex chromosomes. The traditional view of sex chromosome evolution posits that the dosage imbalance in XY males caused by Y degeneration leads to the evolution of a compensatory mechanism called dosage compensation. Their in-depth assessment is that the forces driving the evolution of dosage compensation so far remain elusive, both in plants and animals. Notably, nascent dosage compensation may be causal rather than consequential for the evolution of suppressed recombination between heterogametic sex chromosomes in animals and plants [25]. Y chromosome recombination arrest and degeneration may thus occur without selection related to sexual dimorphism.

### (b) The main pathways to dioecy in seed plants

In gymnosperms, dioecy probably evolved from monoecious ancestors that carried ovulate cones and pollen cones on each individual. One gymnosperm lineage, studied by Gong & Filatov [8], is *Ginkgo biloba* (Ginkgoales) for which the authors also assume ancestral monoecy and derived dioecy, comparing *Ginkgo* to conifers, with 1000 living species in which dioecy has evolved from monoecy at least ten times [8]. In flowering plants, too, monoecy appears to be the most common sexual system from which dioecy evolved [4,26]. Whether unisexual flowers and a monoecious sexual system or instead bisexual (perfect, monoclinous) flowers and a hermaphroditic sexual system are the ancestral condition in angiosperms remains unknown. This is the case because neither the fossil record, which has yielded both unisexual and bisexual Cretaceous flower fossils, nor molecular phylogenies have so far provided an answer. The latter's inability to tell us which sexual system is ancestral will not change since all extant outgroups have unisexual cones (strobili), while bisexual flowers (amphisporangiate strobili [27]) are considered a synapomorphy of angiosperms. *Amborella*, the sister to all remaining living angiosperms, is dioecious with a ZW system [28,29].

Pathways to and from dioecy can be inferred with considerable confidence within smaller clades, with an interesting example provided by the genus *Mercurialis*, one species of which, the dioecious *M. annua*, attracted the attention of the young Linnaeus and is shown on the cover of this special issue. The analyses of Gerchen *et al.* [30] reveal that this widespread species has perennial and annual diploid, tetraploid and hexaploid forms, with the latter two being allopolyploids. Fitting with this patchwork, experimental evolution in *M. annua* has demonstrated that transitions from dioecy to monoecy can occur in just a few generations via the selection of ‘leaky’ sex expression in female plants, following the removal of male plants [31]. Polyploidization is not consistently correlated with breakdowns of dioecy, however.

The genetic basis of plant sex-determining systems may also inform us about the evolutionary pathways to dioecy. From a developmental-genetics perspective, single-gene sex determination appears likely to evolve via monoecy [3]. The epistatic genetic interaction between the feminizing and masculinizing genes essential for monoecy to be expressed is maintained in the dioecious system. On the other hand, recessive male sterility mutations, which are found in two-gene systems of sex determination and often directly function in tapetum or pollen development (e.g. *TDF1* in asparagus, *FrBy* in kiwifruit or *INP1* in grapevine), are much more difficult to reconcile with a path via monoecy. Elucidating the genetic and molecular basis of the sexual systems in closely related monoecious and dioecious species will be a key to confirm these predictions.

### (c) The sizes and trajectories of non-recombining regions with sex-determining genes

The evolution of non-recombining sex-linked regions still poses many questions. Studies of dioecious plants (and gonochorous animals) offer the possibility of testing whether pre-existing recombination deserts, such as the regions around centromeres or other non-recombining haplotype blocks [32], could explain why sex-determining genes are often located within non-recombining regions, without needing to invoke selection and sexually antagonistic polymorphism [2]. In several of the taxa studied in this special issue, such as *Ginkgo*, *Mercurialis* and *Populus*, the non-recombining region with the sex-determining genes is small, and this does not appear to relate to the age of the respective systems, although this is extremely difficult to infer [33].

Even closely related taxa differ in the size of their non-recombining region. Thus, in hexaploid *M. annua*, the non-recombining region appears small, which contrasts with the large sex-determining region inferred for diploid *M. annua* although this inference is so far based on a limited number of individuals and may thus be an overestimate [30]. This difference suggests that the ancestral Y chromosome in the genus *Mercurialis*, which has been diverging in a number of different species, has likely seen differential rates of expansion of its non-recombining regions. In *Rumex*, Rifkin *et al.* [12] provide evidence for sex differences in recombination, with pericentromeric regions of highly suppressed recombination in males that cover over half of the genome. These differences are found on autosomes as well as sex chromosomes, suggesting that pre-existing differences in recombination may have contributed to sex chromosome formation and divergence as suggested by Charlesworth [2]. The region of chromosome 2 around the sex-determining region of *G. biloba* also is fairly small despite an estimated age of the sex chromosomes of 125 millon years, and recombination rates do not exhibit marked differences between XX females and XY males [8].

In stark contrast to these plant groups, the non-recombining regions on the Y chromosomes of *Coccinia grandis* and its newly recognized sister species *C. schimperi* are huge (78 cM on the female meiotic map), and a genetic map for *C. grandis* suggests recombination arrest shortly before or after the two species diverged about 3.6 Ma [10].

### (d) The identification of sex-determining genes and the molecular pathways connecting them to the formation of male and female floral structures

Identifying the sex-determining genes is a key goal of sex chromosome research. Only with the knowledge of genes can we fully understand how sex chromosomes form and evolve. Largely due to new sequencing technologies, especially long-read sequencing, the identification of sex-determining regions and candidate sex determinants is possible at an ever-increasing pace in more and more species. Perhaps not surprisingly, independent sequencing efforts, resulting in different reference genomes and using different mapping populations, can lead to ambiguous results. In this special issue, Gong & Filatov [8] present new data to resolve previous controversies regarding the location and size of the sex-determining region in *G. biloba* and thereby provide a refined list of candidate genes. Working on species that so far lack candidate sex-determining genes, Janousek *et al.* [10] and Rifkin *et al*. [12] use DNA- and RNA-seq to characterize the non-recombining sex-determining regions located on the large heteromorphic Y chromosomes of *C. grandis* and *Rumex hastatulus*, respectively.

Despite the remarkable progress in the elucidation of plant sex-determining regions and genes, knowledge on the molecular mechanisms downstream of the sex determinants ultimately causing the differential development of female or male floral organs is lagging behind. Especially the early stages of differential floral organ formation could reveal the underlying signalling pathways. In *Populus*, Leite Montalvão *et al*. [11] show that the single-gene sex switch ARR17 regulates expression of a narrowly defined genetic network. This network appears to converge on the deeply conserved regulator of floral development *PISTILLATA* (*PI*), which is essential for the formation of male stamens also in plants with monoclinous flowers, such as *Arabidopsis thaliana*.

Sex expression is inherently labile [18]. While secondary traits determined by sex chromosomes, e.g. inflorescence architecture in *Mercurialis* [30], can be largely stable, a certain level of plasticity appears to be the norm for floral organ development. This widespread pleogamy may indicate a multi-layered structure of the respective molecular signalling pathways with multiple points for peripheral inputs (see also [3]). It will be exciting to further flesh out these pathways in different dioecious species and determine their role in the flexibility of plant sexual systems.

## 3. Conclusion and outlook

Current views on embryophyte sex determination have long tended to be generalizations based on a handful of well-studied species and pre-molecular-genetic views from the 1950s. The articles in this special issue incorporate data from additional taxa and new insights from animal sex-determining systems [1–3]. While the four facets of plant sexual systems on which we have focused in this introduction exclude other important areas, such as the turnover of sex chromosomes and the role of sexually antagonistic factors in the evolution of sex chromosomes, we nevertheless hope that the framework provided in table 1 will help researchers build on each other's insights. Understanding the molecular mechanisms of sex determination is of key importance if we are to manipulate seed quality and fruit production because many commercially important species are monoecious or dioecious. The genetic control of the distribution of unisexual cones, or flowers, in populations is therefore not just biologically exciting, but important for modern silviculture and agriculture, from poplars and asparagus to wine and *Cannabis*.

## Data Availability

This article has no additional data.
